# From “*N* of 1” to *N* of more

**DOI:** 10.1101/mcs.a000521

**Published:** 2015-10

**Authors:** Ralph J. DeBerardinis, Elaine R. Mardis

**Affiliations:** 1 UT Southwestern Medical Center, Dallas, Texas 75390, USA;; 2 Washington University School of Medicine, St. Louis, Missouri 63108, USA

The current era of biomedical research finds us surrounded by massive amounts of molecular data—particularly genomic data—both from populations and from individual patients. High-throughput methods to characterize the genome, transcriptome, proteome, and metabolome, combined with innovative approaches that define protein–DNA interactions, epigenetic modifications of DNA and histones, allele specificity, and noncoding RNA expression are producing data at a dizzying pace. The ability to generate genomic data quickly and affordably dominates the motivation toward personalized therapies—taking what we have learned from discovery-based efforts and applying it to improve individual patient care and outcomes. Precision medicine initiatives now endorsed by the White House seek to develop cohorts and deploy the associated “big data” to improve understanding of complex diseases and stimulate new diagnostic tests and treatments.

Along with these large-scale discovery efforts, researchers and clinicians already are using high-content data sets to diagnose and treat individual patients. In this translational paradigm, conventional medical evidence is combined with high-content data from a single patient, leading to actionable, even therapeutic, insights within days. Although each anecdote is initially patient-specific, it is increasingly common to observe situations where data from one patient could inform the care of other individuals with molecular and clinical similarities. It is now a major challenge and an opportunity to develop effective ways to disseminate new data and conclusions about potentially informative patients to clinicians and geneticists worldwide. Indeed, this type of communication is one of the goals of *Cold Spring Harbor Molecular Case Studies.* If our communications are successful, desperate parents of children with undiagnosed conditions will no longer need to embark on diagnostic odysseys like Matt and Cristina Might did (“Hunting down my son's killer,” http://matt.might.net/articles/my-sons-killer/), physicians will be alerted to extraordinary clinical presentations and responses, and basic scientists will be given leads for translational research. In doing so, “*N* of 1” becomes *N* of many. As individuals aggregate into cohorts, pathophysiology and therapeutic opportunities should come into sharper focus.

Effectively, the journal aims to profile precision medicine in action. We project that our content will include not only descriptive case studies but also a forum to add information pursuant to the initial description to report the success or failure of therapeutic interventions. In this regard, we encourage the reporting of information even if the result is “negative” relative to expectation—this is still critically important to share. We consider the manuscripts from David Goldstein's laboratory, both of which are published in this inaugural issue, to be exemplary of the Research Report and Follow-Up Report formats. Our desired breadth spans many medical disciplines—not just oncology and medical genetics but the total spectrum of precision medicine and its associated measurements. Our rapid publication of accepted manuscripts will ensure timely communication of case reports and full-length research articles. Position Statements and Commentaries will highlight emerging applications, challenges, and paradigm shifts in the precision medicine space.

We invite you to explore this inaugural issue, which we feel highlights our goals for the journal and the potential impact of rapidly reporting disease-relevant high-content data sets. We encourage the submission of manuscripts featuring high-content data sets from individual patients, families, and larger cohorts to further the dialog and discourse on these topics and to ensure that pertinent information about precision medicine efforts is widely disseminated in the biomedical research and clinical practice communities.


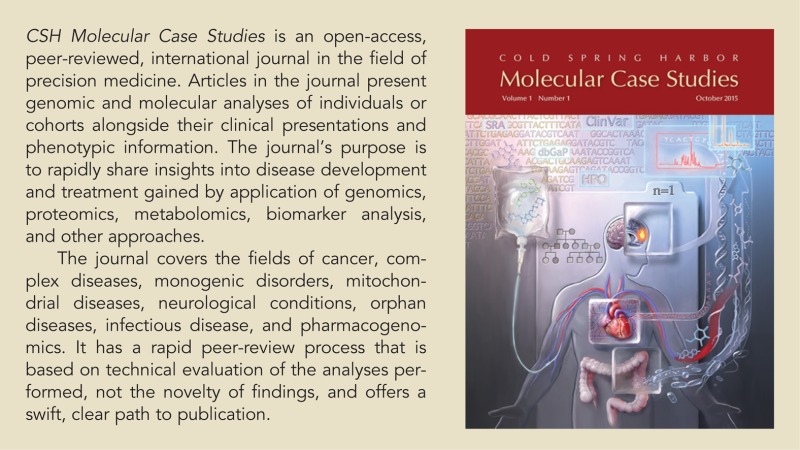


## Competing Interest Statement

The authors have declared no competing interest.

